# A case report of drug interaction between co-packaged nirmatrelvir-ritonavir and tacrolimus causing hyponatremia in a lung transplant recipient

**DOI:** 10.1186/s13019-024-02599-w

**Published:** 2024-03-15

**Authors:** Chien-Ming Lo, Wei-Hsun Chen, Meng-Yun Tsai, Hung-I Lu, Yu-Hsin Hsiao, Kai-Hao Chuang, Yu Chen, Hsuan-Feng Wu, Kuo-Tung Huang, Yi-Hsi Wang

**Affiliations:** 1 Division of Thoracic and Cardiovascular Surgery, Kaohsiung Chang Gung Memorial Hospital, Kaohsiung, Taiwan, ROC; 2 Division of Thoracic and Cardiovascular Surgery, Linko Chang Gung Memorial Hospital, Linkou, Taiwan, ROC; 3 Division of Chest, Kaohsiung Chang Gung Memorial Hospital, Kaohsiung, Taiwan, ROC

**Keywords:** Lung transplantation, COVID-19, Tacrolimus, Drug interaction, Antiviral agent, Nirmatrelvir-ritonavir, Combination

## Abstract

**Background:**

Coronavirus disease 2019 (COVID-19) infection in lung transplant recipients can be lethal owing to the use of immunosuppressants. Antiviral agents may be administered to these patients. Co-packaged nirmatrelvir-ritonavir is a new agent currently being used in combination.

**Case presentation:**

In this report, we present a case of a 64-year-old woman, a lung transplant recipient, who experienced hyponatremia and showed a high serum tacrolimus concentration following the administration of the co-packaged nirmatrelvir-ritonavir combination.

**Conclusion:**

Although the nirmatrelvir-ritonavir and tacrolimus combination is not contraindicated, other treatment strategies should be considered first, if available, and the dose of tacrolimus should be reduced when using the nirmatrelvir-ritonavir combination. In cases where combination therapy is necessary, serum tacrolimus levels should be closely monitored in lung transplant recipients. Documentation of more such reports is important to identify drug interactions between nirmatrelvir-ritonavir and other agents, with the aim of preventing severe adverse effects.

## Background

The outcomes of lung transplant recipients infected with coronavirus disease 2019.

(COVID-19) are poor [1]. To date, an appropriate treatment strategy for COVID-19 infection in lung transplant recipients has not been established [2]. Some small retrospective studies have provided suggestions for lung transplant patients, whereby stopping immunosuppressants such as mycophenolate or mTOR inhibitor and reducing the tacrolimus is the first step. Antiviral agents such as protease inhibitors should not be prescribed for lung transplant recipients owing to drug interactions [3].

Nirmatrelvir-ritonavir is a new co-package containing 300 mg of nirmatrelvir (two 150 mg tablets) with 100 mg of ritonavir (one 100 mg tablet). It received emergency use authorization by the Food and Drug Administration in December 2021 [4]. Nirmatrelvir-ritonavir is an active protease inhibitor that exerts its antiviral efficacy by inhibiting a necessary protease in the viral replication procedure [5]. Oral administration of nirmatrelvir (300 mg) with ritonavir (100 mg) every 12 h for 5 days commencing within 3 days of the onset of COVID-19 symptoms is proven to reduce hospitalization and death [6]. The use of nirmatrelvir-ritonavir in lung transplant recipients is not a contraindication but requires close follow-up due to the administration of immunosuppressants such as tacrolimus.

Taiwan has a low volume of lung transplant cases. Only 20 to 30 recipients receive lung transplant surgery annually nationwide. COVID-19 infection in lung transplant recipients is rare and a treatment protocol has not been established. Herein, we report a case of severe hyponatremia in a lung transplant recipient who was administered nirmatrelvir-ritonavir and tacrolimus. This study was approved by the institutional review board of Chang Gung Memorial Hospital (IRB No. 202201455B0).

## Case presentation

**Fig. 1:**
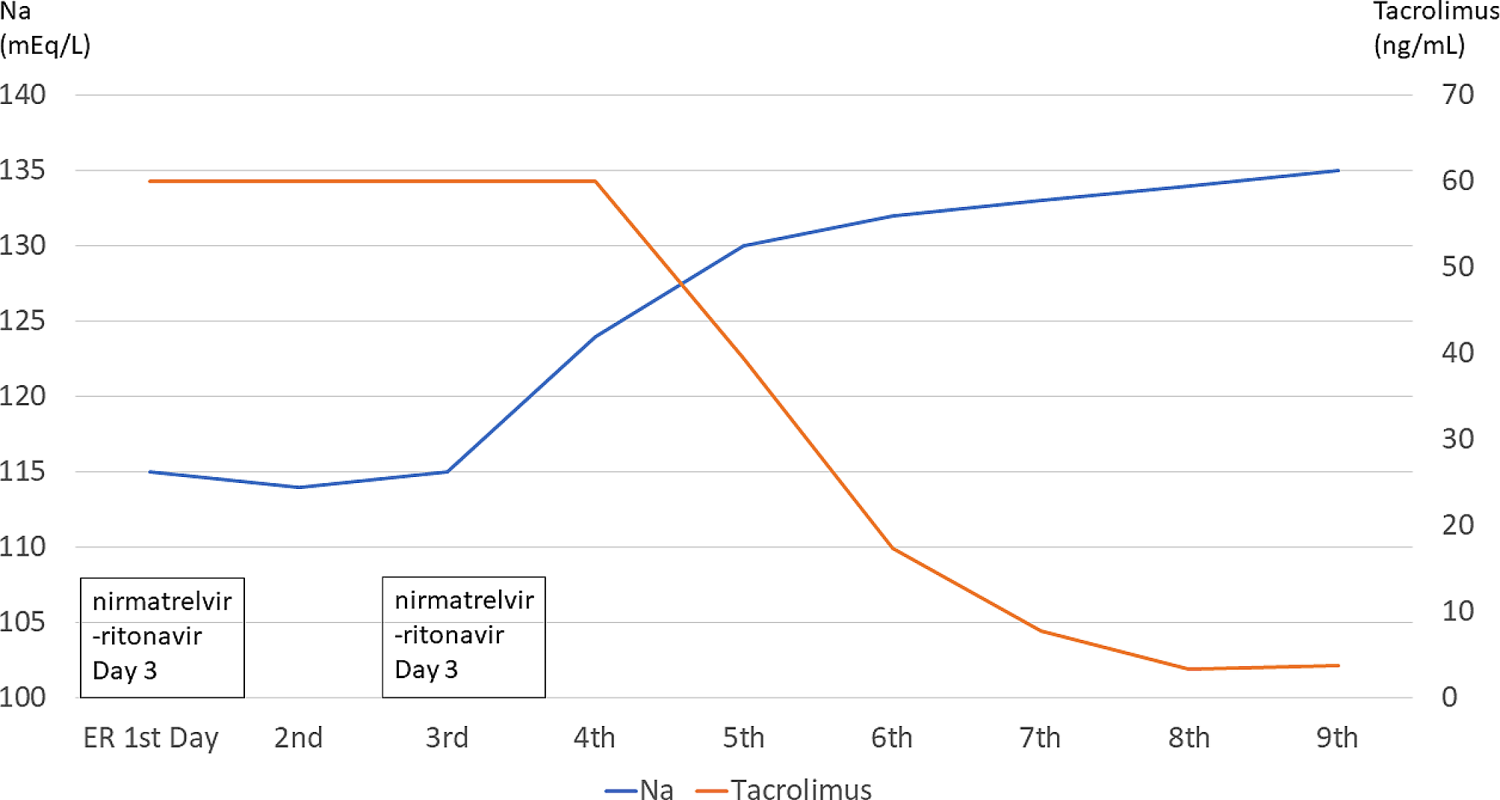
Drug interaction between nirmatrelvir-ritonavir and tacrolimus can cause hyponatremia

A 64-year-old woman underwent bilateral lung transplantation on 2021-10-07 due to bronchiolitis obliterans organizing pneumonia in Kaohsiung Chang Gung Memorial Hospital. She received oral prednisolone (10 mg every 12 h), mycophenolate mofetil (500 mg every 12 h), and tacrolimus (4 mg every 12 h) 3 months before case presentation. She also took sulfamethoxazole-trimethoprim and valganciclovir and inhaled amphotericin B. The tacrolimus trough level was 6 to 8 ng/dL during the 3 months, which is within the goal trough level in our lung transplant program. She did not take any medication which could have led to electrolyte imbalance such as diuretics or selective serotonin reuptake inhibitors. COVID-19 infection was confirmed by rapid antigen testing as she showed mild sore throat symptoms and visited a nearby clinic. She was prescribed nirmatrelvir-ritonavir for treatment. She experienced weakness and was easily fatigued two days after receiving the treatment without other obvious neurologic signs. She visited the emergency department of our hospital and was admitted to a separate ward with other patients with COVID-19 who did not require critical care. The laboratory data showed hyponatremia (115 mEq/L) and elevated tacrolimus levels (> 60 ng/mL) when she visited the emergency department. Other laboratory data were as follows: serum creatinine, 0.56 mg/dL; serum osmolality, 231 mosm/KgH_2_O; urine sodium, 41 mEq/L; urine creatinine, 24.3 mg/dL, and urine osmolality, 220 mosm/KgH_2_O. Fractional excretion of sodium was 0.8%. We stopped tacrolimus and mycophenolate mofetil immediately. The creatinine and potassium data were within normal limits. Owing to a stable condition without other obvious neurologic signs, we did not prescribe any medication or intervention for hyponatremia. We admitted her for close monitoring. After completion of the nirmatrelvir-ritonavir treatment 5 days later, hyponatremia improved gradually, and tacrolimus levels returned to the therapeutic level. We resumed the prescription of tacrolimus (2 mg every 12 h), mycophenolate mofetil (500 mg every 12 h), and prednisolone (10 mg every 12 h) after stopping nirmatrelvir-ritonavir 2 days. She was discharged without any neurologic deficits after 7 days of admission to the emergency department. Laboratory findings during the clinical course are shown in Fig. 1.

## Discussion and conclusion

Nirmatrelvir reduces the duration of hospital stay and mortality in patients with COVID-19 [6]. Ritonavir is a pharmacokinetic enhancer [7, 8]. The combination of nirmatrelvir and ritonavir is a strong inhibitor of CYP3A induced by ritonavir. Degradation of tacrolimus by CYP3A4 and CYP3A5 occurs mainly in the liver [9]. Although tacrolimus is not contraindicated in combination therapies, a drug–drug interaction has been predicted and tacrolimus dose should be reduced [8, 10].

Tacrolimus can induce hyponatremia in lung transplant recipients but its mechanism is not fully understood [11]. One possible theory is that tacrolimus damages distal renal tubules and causes type IV renal tubular acidosis. However, our patient did not show kidney injury even during close monitoring of her creatine level every 8 h upon admission. Banks et al. suggest that some patients can maintain normal kidney function even under hyponatremia. The treatment of hyponatremia is challenging owing to its unclear etiology and multifactorial nature [11]. If neurologic symptoms are obvious, fluid restrictions and salt supplementation can be helpful. However, our patient was stable and showed no neurologic deficit. Her weakness improved after stopping tacrolimus. We hypothesize that our patient experienced drug–drug interaction between nirmatrelvir-ritonavir and tacrolimus which led to a high trough level of tacrolimus, which eventually induced hyponatremia.

In conclusion, nirmatrelvir-ritonavir combined with tacrolimus can cause high serum tacrolimus levels and intoxication. Hyponatremia and acute kidney injury may develop following combination therapy. If combination therapy is necessary, serum tacrolimus levels should be closely monitored. If hyponatremia develops due to tacrolimus without obvious neurologic signs, aggressive correction of hyponatremia may not be necessary. More such reports are needed to identify drug interactions between nirmatrelvir-ritonavir and other agents to prevent severe adverse effects. Although the combination of nirmatrelvir-ritonavir and tacrolimus is not contraindicated, other treatment options should be considered first, if available. The dose of tacrolimus should be reduced for nirmatrelvir-ritonavir combined usage.

## Data Availability

All data generated or analyzed during this study are included in this article.

## Abbreviations

COVID-19: Coronavirus disease 2019
